# The Effect of Kinesio Taping on Handgrip and Active Range of Motion of Hand in Children with Cerebral Palsy

**Published:** 2017

**Authors:** Zabih ALLAH RASTIl, Alireza SHAMSODDINI, Hamid DALVAND, Sina LABAF

**Affiliations:** 1 Exercise Physiology Research Center, Baqiyatallah University of Medical Sciences, Tehran, Iran.; 2 Department of Occupational Therapy, Arak University of Medical Sciences, Arak, Iran.; 3 Department of Occupational Therapist, Ebnesina Rehabilitation Clinic, Consulting Unit, Tehran, Iran.

**Keywords:** Cerebral palsy, Kinesio taping, Grip strength, Range of motion

## Abstract

**Objective:**

Kinesio taping is a relatively new technique, which uses in rehabilitation of neurologic diseases. The aim of this study was to investigate the effects of Kinesio taping on hand grip and active range of motion of hand in children with cerebral palsy (CP).

**Materials and Methods:**

In this experimental study with pre-test and three post-tests, 32 children with CP randomly were placed in experimental (n=17) and control group (n=15).Kinesio taping was applied on dorsum of forearm and hand. Evaluation was performed initially, two days after taping and two days after tape removal. Goniometer was used to evaluate active range of motion of wrist extension. In addition, vigorimeter was used to evaluate of grip strength.

**Results:**

In pre-test, there was no difference between groups but in post-tests; initially after application of taping with *P*<0.05, two days after application of taping with *P*<0.05 and follow-up (two days after removed taping) with *P*<0.05 were significant differences between trial and control group.

**Conclusion:**

Kinesio taping in neurorehabilitation of children with CP can be a useful option to promote power or grip strength and active range of motion of wrist and thumb.

## Introduction

Cerebral palsy (CP) resulting from damage to non-mature brain that occurring before, during, or after birth and causes permanent disorder of movement and posture (1). The classification of CP is topographically (hemiplegia, diplegia, and quadriplegia) or based on motor function as spastic and non-spastic (including athetoid, ataxic and Dystonic). CP prevalence per 1000 live births is about 2 to 2.5 (1, 2). CP leads to spasticity, intensify reflexes, co-contraction, weakness or loss of movement control, muscle weakness, defects in sensory integration, lack of muscle coordination, balance and postural control limitation (3, 4). 

Neurological lesion in CP is non-progressive, but musculoskeletal lesions is progressive (5). Secondary musculoskeletal effects like muscle weakness or muscle imbalance, pain, restriction of active and passive range of motion and poor functional use of limbs can be seen (6). 

Muscle deficiency in CP can lead to limitations in range of motion, accurate timing, power and hand manipulation skills (7). One of the main challenges in children with CP is dysfunctional of hand which interference in use of hands and limits the child's ability in activities of daily living, communication and social contacts (8, 9). Neurologic deficits such as spasticity, co-contractions, muscle weakness and limited range of motion have negative effects on dexterity and functional use of hands (10). 

The most common upper extremity posture in children with CP include internal rotation of the shoulder, elbow flexion, internal rotation of the forearm, wrist flexion, fingers flexion, and thumb in palm(11). Wrist position can affect the grip and hands strength so that in wrist flexion (common hand problem in cerebral palsy) reduction in hand strength is significant. 

The American Society of Hand Therapists noted that in 0 to 35 degrees of wrist extension, strength is greater than other positions (12-14). Grip, pinch, and dexterity are the main functions of hands and support the daily activities; if these performances are disrupted due to neuromuscular disorders such as CP, child’s performance is reduced in activities such as homework, self-care and the interact with peers (7). Hand function is necessary to interact with the environment and discovering the world. Hands provide performance through contact with people and objects; and are a tool used many times for play, work and perform activities of daily living, (15).

International classification of functioning system (ICF) explains that CP affects the body structures (e.g. limbs), body function (e.g. intellectual function), activities (e.g. standing/walking) and participation (e.g. sport). “Psychomotor disorders in children with CP results in limitation in use of the limbs, more paralysis, difficulty in performing activities of daily living (ADL), more dependence and ultimately lower quality of life(16, 17). 

Many therapeutic methods are used to treating the sign of CP including botulinum toxin injection(18), orthopedic surgery, constraint-induced movement therapy (CIMT), medications (19), occupational and physical therapy (20-23). Occupational and physical therapy use various dynamic approaches including Bobath (24), sensory integration (SI), proprioceptive neuromuscular facilitation (PNF) (25) and the Brunnstrom techniques (1, 24) to adjust the muscle tone**, **reduce contractures, improve the range of motion (ROM), improve the sensory and cognitive problems, improve muscles strength hand fostering children's independence level in ADL (26-28). 

A new method recently used for reducing problems of hand and upper limb in children with cerebral palsy is Kinesio taping (KT). Although, KT initially was used in sport or orthopedic fields and then approved as an adjunctive treatment in other functional impairments such as neurological disorders (6, 29-31). 

KT is latex-free with 100% cotton fibers that has no pharmaceutical effect (32), designed to mimic the elasticity properties of the muscle, skin, and fascia (31). Skin, lymphatic system, circulatory system, fascia, muscle, and joint can influence by KT (33). KT can cause enhancing proprioception (34), diminishing pain and edema, reducing muscle spasms, and strengthening the muscles (35, 36). KT can effect on muscle performance and support joint by improving proprioception, normalizing muscle tone, correct the inappropriate positions and stimulate skin receptors (4, 37-39). KT is used for strengthening weak muscles and improves joint stability and alignment to influence positively use of hand in task(6, 37, 38). 

Although, the use KT with pediatric populations is not well studied, but studies show KT when applied in adjunct with traditional therapy interventions such as stretching, neurodevelopmental therapy, practicing functional tasks can further improve recovery in children with CP (40). This study aimed to determine the effect of KT on handgrip and active range of motion (AROM) on hand in children with CP.

## Materials& Methods

***Subjects***

Based on inclusion criteria, thirty-two subjects with CP were randomly selected from the Occupational Therapy Clinic in Tehran, Iran. Subjects were divided into two equal groups (trial and control group). They were between 4 and 14 yr of age. Inclusion criteria including: 1- children with cerebral palsy, confirmed by pediatric neurologist, 2- Hand and/or wrist spasticity less than three according to Modified Ashworth Scale (MAS), 3- have thumb in palm and wrist flexion deformities without upper extremity passive range of motion limitation, 4- Sufficient cognitive level to follow the directions of the testing protocols and tape acceptance. Exclusion criterion was allergy to taping.

This study was approved by the human research Ethics Committees of the Baqiyatallah University of Medical Sciences, Tehran, Iran. An informed consent was obtained from all parents of subjects, including agreement of the children to participate as volunteers. 

For evaluation of grip strength and AROM, we used Vigorimeter (A common tool in hand therapy, which measures the grip strength by pressing the rubber bulb). It has three rubber bulbs in different sizes that measure power in terms of kilopascal (kPa) (41, 42). We used these means to assess the power grip (a grasp with opposed thumb and flexed fingers (43) by pressing the large bulb with all fingers. Testing performs in standard posture suggested by the American Society of Hand Therapists for grip strength tests: sitting on a chair with no armrest, shoulder adducting and rotating to the neutral position, 90° elbow flexion, and forearm and the wrist joint in the neutral position (44). Children do it three times and finally, the average of these three attempts was recorded as the final number. 

Goniometer: was used to evaluate AROM of wrist extension (45). To measure the range of motion, children sat on a chair with a standard height and standard desk in front. Then, for wrist extension, fisted hand with middle position of the forearm were placed on the table and asked child to fully extend him/her wrist and hold in the end of range. Axis of goniometer placed above the radial styloid process in the snuffbox, stationary arm placed parallel to radius bone and moveable arm parallel to metacarpal bone of index finger and then the number to be recorded (46). To examine thumb extension for measuring the thumb extension, palm of hand was placed on the table (full forearm pronation with the full adduction of all fingers). Then asked child’s too far out the thumb from other fingers, fully (47-49).

**Procedure**

**Fig 1:**
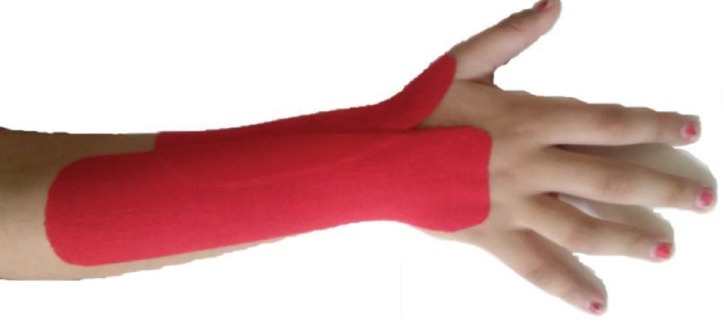
Kinesio Taping used in this study

In this experimental study with pre-test and 3 post-tests (initially and 2 d after taping, 2 d after tape removal), 32 children who had the inclusion criteria, randomly were placed in experimental and control group (17 in experimental group and 15 in control group). This study was performed in *Tehran*, Iran from 2015 to 2016. In the beginning of the study and after taking pre-test, KT applied as follows: in intervention group, from origin of extensor digitrumcombines muscle to metacarpophalangeal (MP) joint of fingers; and from origin of extensor and abductor policieslongs to MP joint of thumb. Tension of tape in muscular zone was 30% and in joint area was 75% (Figure1). Purpose of KT application in these manners was to improve the function of muscles and joint re-alignment (correction the wrist flexion and thumb in palm deformities). In control group, KT was used as placebo in similar method with another group but with no tension and as sham. Evaluations were taken immediately and two days after application of taping and then KT was removed from the hand. Two days after KT removal, testing was done again.

**Statistical analysis**

The normal distribution of variables was assessed with the Kolmogorov-Smirnov test. An independent sample *t*-test was used for comparison of age and gender between the two groups. Analysis of Variance (ANOVA) was used to identify between groups differences. Statistical analysis was performed with SPSS ver. 17.0 (Chicago, IL, USA), with *P-*values less than 0.05 as statistically significant.

## Results

Only two subjects in trial group didnot succeed to complete the protocol due to delay in referral for follow up assessment. Thirty children with CP participated in this study; 15 subjects in trial group (age range 5-14 yr, mean age 8.33 yr) and 15 subjects in control group (age range 4-13 yr, mean age 7.4 yr). Comparison of age and gender between two groups were done with independent sample *t*-test. There were no significant differences between groups with *P*=0.31 and *P*=0.072, respectively. Table1 shows some descriptive information of groups. Descriptive and analytic statistics for grip strength and AROM (wrist extension and thumb extension/abduction) of two groups (Trial and Control) in pre-test, post-tests, and follow-up situations are shown in Table 2 and 3. In all variable, there wasno difference between groups but in post-tests (initially, 2 d) and follow-up (2 d after removed taping) were significantly differences (Table 2 and 3).

**Table 1:** Descriptive statics in the trial and control groups

	Mean age	Gender		Affected hand		CP type		
		M	F	R	L	Hemiplegia	Diplegia	Quadriplegia
Trial group	8.33	5	10	11	4	5	3	7
Control group	7.40	10	5	11	4	6	1	8

M; male, F; female, R; right, L; left

**Table 2:** Comparison of AROM between groups

	Wrist extension			Thumb extension			Thumb Abduction		
	Mean±SD		*P*_-value_	Mean±SD		*P*_-value_	Mean±SD		*P*_-value_
	Trial	Control		Trial	Control		Trial	Control	
P0	28.87±7.51	23.07±11.7	0.1	26.53±10.56	20.93±11.68	0.18	28.33±11.83	21.07±11.62	0.1
P1	37.67±7.41	25±11.13	0.001	36.87±8.32	22.6±10.56	0.000	34.8±11.37	21.73±10.51	0.003
P2	37.47±6.08	24.27±10.91	0.000	37±8.12	22.87±10.27	0.000	36.73±8.16	21.87±10.53	0.000
P3	34.07±6.99	23.2±11.16	0.003	32.87±10.5	23.2±10.35	0.01	32.67±11.31	22.47±10.67	0.01

SD; standard deviation, P0; pre-test, P1; post-test 1, P2; post-test2, P3; post-test 3

**Table 3:** Comparison of Grip strength between two groups

		P0	P1	P2	P3
Mean±SD	Trial	12.07±7.72	16.07±9.15	18.47±10.04	14.4±8.19
	Control	8.8±5.82	8.8±5.77	8.4±5.66	8.47±5.69
*P*_-value_		0.201	0.015	0.002	0.029

SD; standard deviation, P0; pre-test, P1; post-test 1, P2; post-test2, P3; post-test 3

## Discussion

The ROM is one of the essential parts of the body function and structure essentially in hand (50). Secondary contracture and joints deformity can reduce ROM. Flexor carpi ulnaris in children with cerebral is twice more rigid than healthy children that cause stiffness, movement disorder, and hand mal-posture. Hand flexion in CP is dominant which makes problem for AROM of wrist extension (51).

Grip strength is the force generated by the thumb and other fingers required in different functional activities of daily living (44). An objective index assesses functional integrity of the upper extremity (14). 

CP makes muscle weakness (or muscle imbalance) and restriction in active ROM, that could be a barrier to the functional use of hands (6, 7, 9, 14). In children with CP, wrist flexion reduces grip strength about 60%. Hand orthosis can significantly correct this deformity and promote wrist stability, but despite these benefits are unable to restore and enhance grip strength. Many children because of the appearance of device are not willing to accept hand orthosis (52).

In this study, we used KT to correct the wrist flexion deformity in children with CP. When the tape is applied properly, the flexibility of KT not only does not restrict ROM of soft tissue but also supports weak muscles and provides joint mobility (53). 

We applied KT on dorsum aspect of wrist and forearm to support activation of extensor muscles of wrist and thumb and placed wrist in functional position. Our result showed that AROM in all testing joint (wrist extension, thumb extension/abduction) and in all post-tests has been affected by KT; and Child's ability significantly improved in active thumb extension/abduction and wrist extension. We had two post-tests with KT and one without KT (follow-up). Post-test was significantly different; therefore, KT effects remained after it was removed (post-test 3). 

The result of our study in grip strength showed that similar to AROM, KT significantly improves handgrip of children with CP. Imbalance between wrist flexor muscles (spastic) and wrist extensor muscles (weak) in children with CP leading to abnormal posture of hand that affects the ability to grasp (54) and wrist position can affect the grip and hands strength (12-14). KT can correct the abnormal posture of hand, put it in functional position via improving range of motion and stimulation wrist extensors muscles, and inhibit wrist flexors muscles (51). 

The purpose of the application of KT in rehabilitation of children with CP are muscular facilitation or inhabitation, the re-alignment the joints, improve proprioception and postural support (55, 56). Use of this technique provides a tactile-proprioceptive stimulation that facilitates muscle performance.

KT stimulates the skin mechanoreceptors through pressure and stretching that this stimulation may be due to physiological changes such as enough firing in muscle recruitment patterns. Increased use of muscle motor units is the result of proprioceptive stimulation that improves motor performance (4, 57). Moreover, dysfunctional muscle control (defect in neural control) in children with CP leads to tenodesis phenomena in extensor muscles (antagonist). Stability and control of wrist and proximal joints be affected by these phenomena, hence children use their fingers for mass grasp. Thus, if joint control of wrist and forearm is provided, functional control at thumb and fingers will become better, which may facilitate improved functional hand skills and grip strength (58).

The effect of KT on handgrip strength (KT was used on the anterior surface of the forearm) of 75 healthy women aged 18-30 yr old. The results showed a significant increase in grip strength in 30 min, 24 h and 48 h after the KT application (59). 

In another study, KT was applied on the wrist extensor muscles of 15 children with CP to measure the wrist extension AROM. The results were in accordance with our results and AROM of wrist extension (with and without functional ball grasping) were significantly improved (*P*<0.05) (51). 

Immediate effect of correction KT was assessd on hand span in children with CP (60). In that study, KT was applied to help wrist extension. Goniometer was used to measure the wrist extension and a ruler to measure hand span (from index finger to thumb, with hand wide open) (60). All results of these studies were similar to our results and the mean of both variables was significantly increased (51, 59, 60). 

Although in a study, the opposite results were reported, approximately. After using KT on extensor surface of forearm, the AROM of wrist extension was increased but statistically were not significant (61). The effect of KT was investigated on maximum grip strength in 22 healthy athletes’ student. Evaluation was performed in three conditions (without KT, placebo KT, KT). In this study, no significant differences between three conditions were seen (62). 

In Iran, the effects of KT on grip strength in three areas were measured. Participants were healthy students and KT application was done on the extensor, flexors and extensors/flexors to determine the best mode. In all three modes, KT could increase grip strength, but application in extension provides more grip strength than the other two modes (flexors, extensors/flexors) (63). When KT apply without tension (as placebo), not provide the necessary stimulation; and this stimulate is not comparable to KT with proper tension (62). 

We used KT without tension in control group and there was no significant difference in results from these group and could not interfere with the result, so our results were in the same direction; KT without tension (as sham) is not effective.

**In conclusion**, KT in neurorehabilitation of children with CP can be a useful option to promote power or grip strength and AROM of wrist and thumb. Therefore, KT provides easy and effective way to improve function in children with CP and neuromotor impairments.
